# The effects of bathing in neutral bicarbonate ion water

**DOI:** 10.1038/s41598-021-01285-4

**Published:** 2021-11-08

**Authors:** Tomoe Yamazaki, Ryoko Ushikoshi-Nakayama, Supriya Shakya, Daisuke Omagari, Naoyuki Matsumoto, Chiyoko Nukuzuma, Tomoko Komatsu, Masaichi Chang-il Lee, Hiroko Inoue, Ichiro Saito

**Affiliations:** 1 Department of Pathology, Tsurumi University School of Dental Medicine, 2-1-3 Tsurumi, Tsurumi-ku, Yokohama, Kanagawa 230-8501 Japan; 2 Kanagawa Dental University Graduate School of Dental Medicine, Yokosuka-Shonan Disaster Oral Health Research Center & Oxidative Stress/ESR Laboratories, 82 Inaoka-cho, Yokosuka, Kanagawa 238-8580 Japan; 3 Division of Dentistry for the Special Patient, Department of Critical Care Medicine and Dentistry, Kanagawa Dental University Graduate School of Dental Medicine, 82 Inaoka-cho, Yokosuka, Kanagawa 238-8580 Japan; 4 THERMOCELL Clinic, Tokyo Design Center 2F, 5-25-19 Higashi-Gotanda, Shinagawa-ku, Tokyo, 141-0022 Japan; 5 Department of Pharmaceutical Sciences, Nihon Pharmaceutical University, 10281 Komuro, Ina-machi, Kitaadachi-gun, Saitama, 362-0806 Japan

**Keywords:** Physiology, Circulation, Blood flow, Health care

## Abstract

Percutaneously absorbed carbon dioxide enhances blood flow. The mechanism by which it does so is unclear, but we hypothesized that it involves bicarbonate ions. BALB/c mice were bathed in neutral bicarbonate ionized water (NBIW) and showed increased blood bicarbonate levels and blood flow via phosphorylation of peripheral vascular endothelial nitric oxide synthase (eNOS) and production of nitric oxide (NO). Phosphorylation of eNOS and NO production were also increased in human umbilical vein endothelial cells cultured in medium containing NBIW, and NBIW showed reactive oxygen species scavenging activity. In a double-blind, randomized study in men and women aged 30 to 59 years with subjective cold intolerance, bathing in NBIW elevated body temperature faster than bathing in a control solution and improved chills and sleep quality. Taken together, our results show that percutaneously absorbed carbon dioxide changes to bicarbonate ions, which act directly on endothelial cells to increase NO production by phosphorylation of eNOS and thus improve blood flow.

## Introduction

Hot-spa therapy, i.e., bathing in mineral-rich hot water, is popular in many regions of the world and is believed to improve mental and physical discomfort and help maintain good health. Improving blood circulation and elevating body temperature are known not only to prevent circulatory disorders^1^ but also to cure trauma^2^. Recent studies showed that hydrogen sulfide in sulfurous mineral water is diffused into the body via the skin and mucosa^3^. In the body, hydrogen sulfide has antioxidant effects and enhances vascular circulation by vasodilation that is dependent on adenosine triphosphate-sensitive potassium (KATP) channels^4,5^. The potential of hydrogen sulfide to prevent age-related diseases and support treatment of cardiovascular diseases has been attracting attention^6,7^.

Carbon dioxide (CO_2_)-enriched spas, which have been used since the Roman era for recuperation and treatment, are found abundantly all over the world. Many studies^8–12^ found that percutaneous absorption of carbon dioxide has therapeutic effects by promoting blood flow and vascular regeneration in vascular disorders, such as peripheral capillary regression in patients with diabetes^13^ and severe limb ischemia in patients with peripheral arterial disease, and in skin diseases^14^. CO_2_ bathing induces vascular endothelial growth factor (VEGF) synthesis and nitric oxide (NO)-dependent capillary angiogenesis at the site of ischemia^15^. Furthermore, percutaneous absorption of CO_2_ increases mitochondria in skeletal muscle and induces switching of muscular fibers; therefore, percutaneously absorbed CO_2_ was assumed to enhance muscle strength^16^ and was expected to be useful for rehabilitation in older people, after operations, and in sports medicine.

The mechanism by which percutaneously absorbed CO_2_ enhances blood flow was previously thought to be attributable to the Bohr effect^17^, in which the absorbed CO_2_ increases CO_2_ pressure at subcutaneous capillaries, causing O_2_ to be released from hemoglobin. However, many aspects of the actual mechanism remain to be elucidated.

When dissolved in water, CO_2_ usually exists as dissolved inorganic carbon (DIC), which is transformed into 3 types—carbonic acid, bicarbonate ions, and carbonate ions—depending on the pH of a solution^18,19^. Carbonic acid is the most prevalent DIC type in weakly acidic solutions, bicarbonate ions in neutral to weakly alkaline solutions, and carbonate ions in alkaline solutions^18^. CO_2_ is percutaneously transferred from the skin surface to the capillary vessels via the transappendageal route, i.e., through appendages such as hair follicles and sweat glands. Blood has a neutral pH range, so bicarbonate ions are the most prevalent DIC type in blood^20^. Therefore, we evaluated the possibility that much of the CO_2_ transferred from the skin to the blood would have a biologically beneficial, physiological effect by forming bicarbonate ions. In fact, one example of direct action of bicarbonate ions on cells is increased antibacterial activity because, when cultured in a medium containing more sodium bicarbonate than usual (which ionizes into bicarbonate ions and sodium ions in an aqueous solution), macrophage cell lines stimulated by lipopolysaccharide and interferon gamma showed enhanced NO production^21,22^. In addition, Buckley et al. demonstrated in children with single-ventricle physiology that bolus injection of NaHCO_3_ enhanced cerebral blood flow in a dose-dependent manner^23^.

On the basis of the above findings, we hypothesized that bicarbonate ions act directly on endothelial cells to enhance NO production in the blood, which improves vascular flow via vasodilation. Therefore, we investigated the effect of bathing in neutral bicarbonate ion water (NBIW) in mice, human umbilical vein endothelial cells (HUVEC), and humans.

## Results

### Effect of NBIW bathing in mice

**Figure 1:**
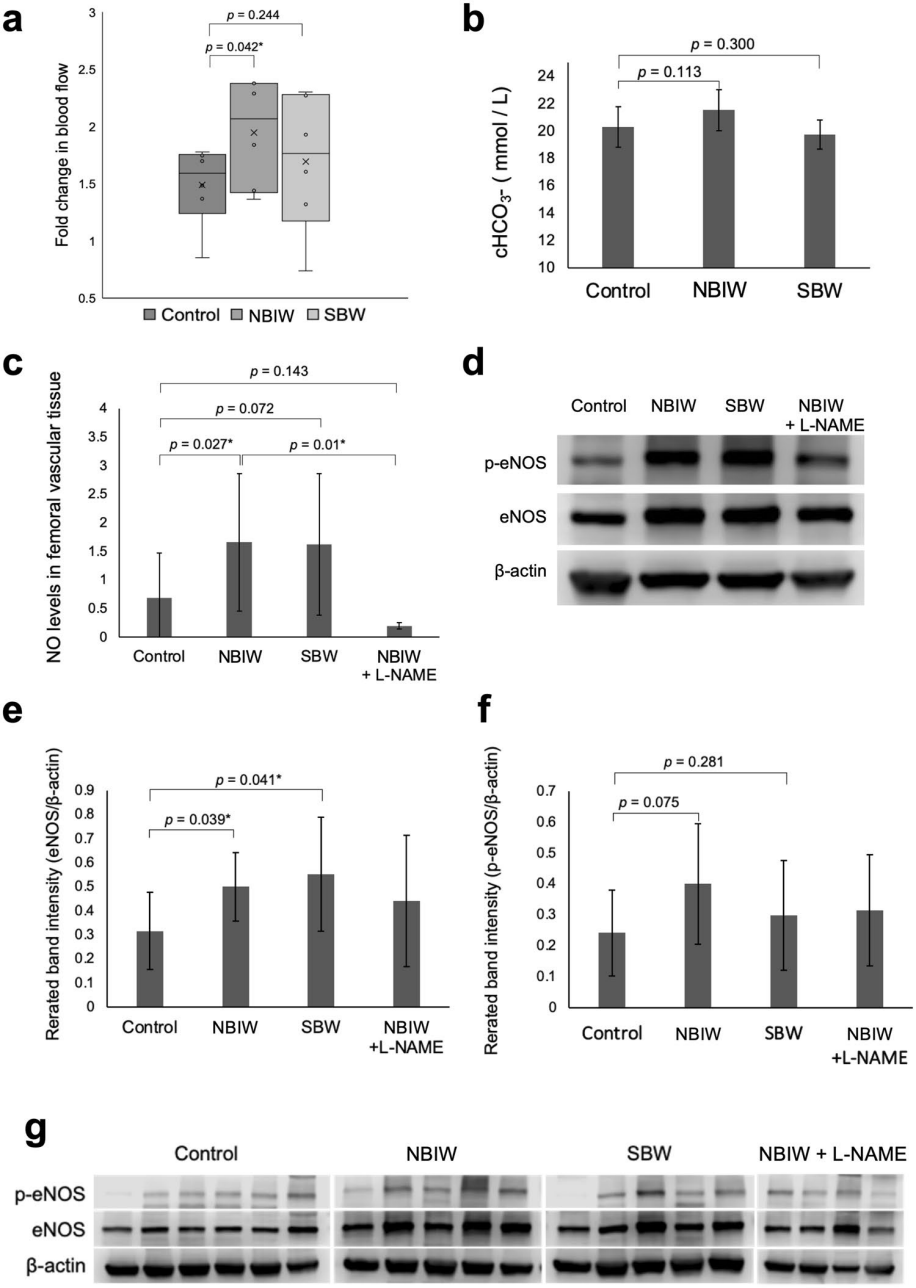
Effects of neutral bicarbonate ion water in vivo. (**a**) Fold change in blood flow in BALB/c mice after bathing. Flow was higher in the neutral bicarbonate ionized water (NBIW) group than in the control group (*p* = 0.042*). (Control: ultrapure water bathing, *n* = 6; NBIW, *n* = 6; SBW: sodium bicarbonate water bathing, *n* = 6). (**b**) Blood bicarbonate ion level after bathing. NBIW:* p* = 0.113 vs control, Hedge’s *g* = 0.83 (95% CI, − 0.46 to 2.124); SBW:* p* = 0.300 vs control, Hedge’s *g* = − 0.44 (95% CI, − 1.892 to 1.003). (Control: *n* = 5, NBIW: *n* = 5, SBW: *n* = 3). (**c**) Level of nitric oxide (NO) in femoral vessel tissue after bathing. The increase was significantly higher in the NBIW than in the control group^†^ (*p* = 0.027*). L-NAME (100 mg/kg) significantly suppressed the NBIW-associated NO increase (*p* = 0.01*). (**d**) Representative Western blot analysis of endothelial nitric oxide synthase (eNOS) and phospho-eNOS (p-eNOS) in a blood vessel in each group (protein carried per lane: 20 μg) in samples from all animals. The β-actin blot is from a different part of the gel blotted with eNOS. Blot images of all animals^†^ are shown in the Supplementary Information. (**e**) The eNOS level in the femoral vessel tissue was significantly higher in the NBIW and SBW groups than in the control group^†^ (NBIW:* p* = 0.039*, SBW:* p* = 0.041*). (**f**) The p-eNOS level tended to be higher in the NBIW and SBW groups than in the control group (NBIW,* p* = 0.075;* d* = 0.95; 95% CI, − 0.299 to 2.205; SBW,* p* = 0.281;* d* = 0.36; 95% CI, − 0.833 to 1.56) and was numerically higher in the NBIW group than in the SBW group. (**g**) Western blot images of all tested animals. ^†^(**c**–**g**) Control: *n* = 6, NBIW: *n* = 5, SBW: *n* = 5, NBIW + L-NAME: *n* = 4. For (**a**,**b**,**e**,**f**) Student's t test was used, and for c, Mann–Whitney U Test (* p < 0.05).

All animals were included in the analysis. NBIW was prepared by adjusting sodium bicarbonate and citric acid to obtain a solution containing bicarbonate ions at 2500 mg/L under pH 7.4. Mice under anesthesia that were bathed in NBIW at 37 °C for 20 min (n = 6) showed significant fold changes in blood flow from before to after bathing compared with control mice (n = 6) bathed in ultrapure water (*p* = 0.042*). On the other hand, mice bathed in sodium bicarbonate water (SBW; n = 5) showed nonsignificant changes compared with control mice (*p* = 0.244) (Fig. 1a). The bicarbonate ion concentration in the blood after bathing tended to be higher in the NBIW group than in the control group but tended to be lower in the SBW group than in the controls (NBIW: *p* = 0.113, Hedge’s* d* = 0.83 [95% CI − 0.46 to 2.124]; SBW: *p* = 0.300, Hedge’s* d* = -0.44 [95% CI: − 1.892 to 1.003]) (Fig. 1b). The amount of NO in the homogenized femoral vessel of the mice was significantly higher in the NBIW group than in the control group (*p* = 0.027^*^) and numerically higher in the SBW group than in the control group (*p* = 0.244). To clarify the involvement of NO, we administered an intraperitoneal injection of L-NAME, an inhibitor of nitric oxide synthetase (NOS), to a group of mice (n = 4) before NBIW bathing and found that the amount of NO in the vascular homogenate showed a significantly greater decrease than in the NBIW group (*p* = 0.01*) (Fig. 1c). Western blotting analysis showed a significant increase in the amount of eNOS in the vascular tissue in both the NBIW and SBW groups compared with the control group (NBIW: n = 5, *p* = 0.039*, SBW: n = 5, *p* = 0.041*; control: n = 6) (Fig. 1d,e). The phospho-eNOS level tended to be higher in both the NBIW and SBW groups than in the control group, whereby the effect size was greater in the NBIW group (NBIW: *p* = 0.075, Hedge’s *g* = 0.95 [95% CI: − 0.299 to 2.205]; SBW: *p* = 0.281, Hedge’s *g* = 0.36, 95% CI [− 0.833 to 1.56]) (Fig. 1f).

The measurements of the pH, PCO_2_, and bicarbonate volume of the NBIW used in the study showed no significant changes from before to during and after the study (see Supplementary Information).

### In vitro experiment in HUVEC

**Figure 2:**
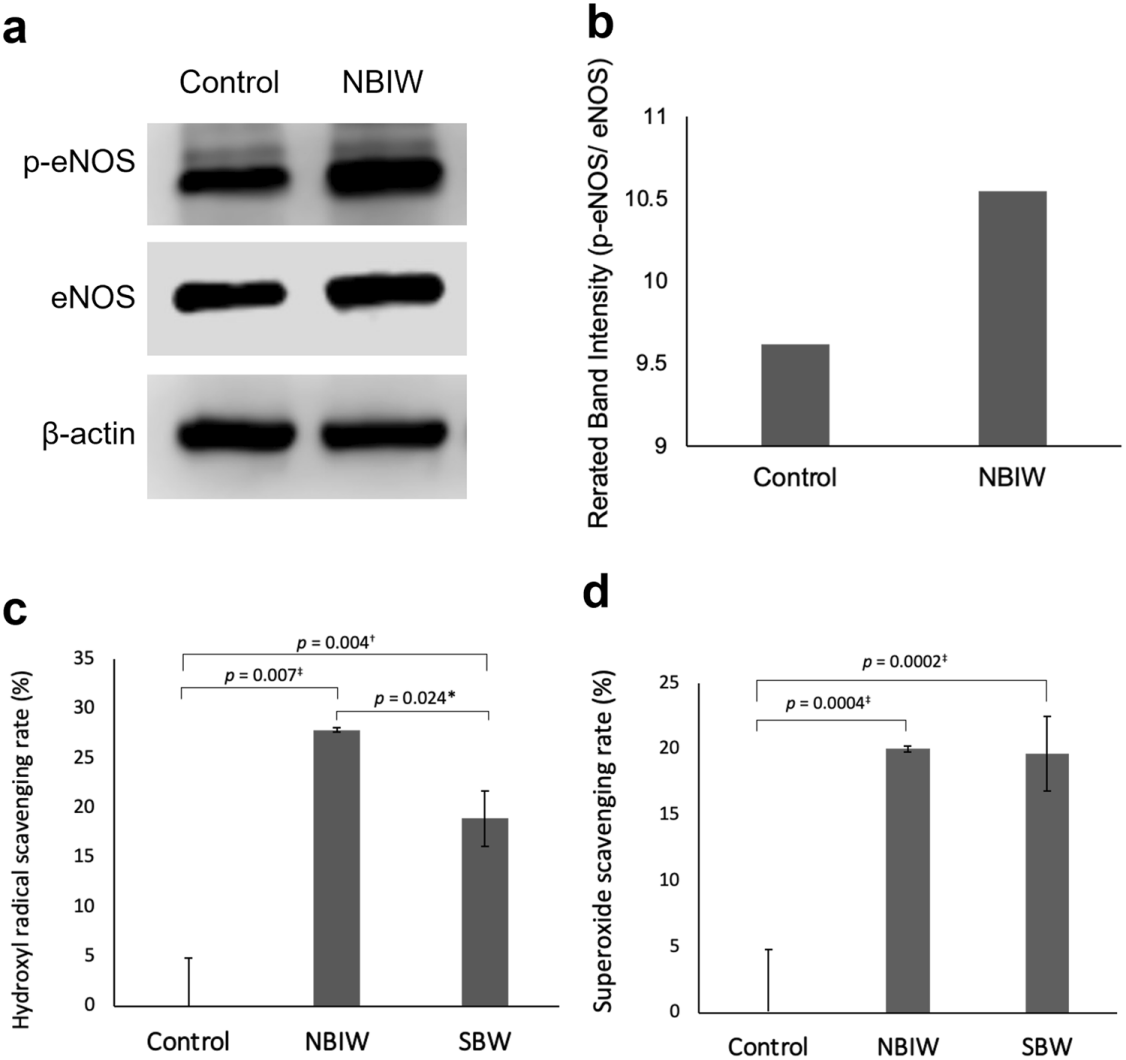
Effect of neutral bicarbonate ion water in human umbilical vein endothelial cells. (**a**,**b**) Effect of neutral bicarbonate ion water (NBIW) in human umbilical vein endothelial cells (HUVEC). HUVEC were serum starved for 6 h, NBIW equivalent to a bicarbonate ion level of 2.5 ppb was added in culture medium, and the cell lysate was analyzed 5 min later. (**a**) Typical example of Western blotting showing endothelial nitric oxide synthase (eNOS) and phospho-eNOS (p-eNOS) levels. The protein carried per lane was 20 μg. The β-actin blot is from a different part of the gel blotted with p-eNOS. eNOS was reblotted onto the membrane blotted with p-eNOS. The full blot is provided in the Supplementary Information. (**b**) eNOS phosphorylation level in cells was higher in cells exposed to NBIW than in those exposed to the control solution. (**c**,**d**) Reactive oxygen species (ROS) scavenging activity of NBIW. Hydroxy radical scavenging ability of NBIW was determined by electron spin resonance (ESR). The mean (*SD*) scavenging rate for a hydroxy radical generated by ultraviolet irradiation/hydroxy peroxide solution was 0.0 (4.85) for ultrapure water (control), 27.9 (0.21) for NBIW, and 18.9 (2.81) for sodium bicarbonate water without citric acid (SBW). The mean (*SD*) superoxide scavenging rate was 0.0 (2.61) for control, 20.0 (0.66) for NBIW, and 19.7 (1.74) for SBW. Each condition was repeated 3 times and analyzed by Student’s or Welch's t-test. *p < 0.05, ^†^*p* < 0.01, ^‡^*p* < 0.005.

To clarify the effect of bicarbonate ions on vascular endothelial cells, we cultured HUVEC in a medium with NBIW that contained bicarbonate ions at 2.5 ppb. After 5 min of culture, the eNOS phosphorylation level was increased (Figs. 2a,b).

### Reactive oxygen species scavenging activity of NBIW

Figures 2c,d show the analysis results of electron spin resonance (ESR) performed to assess the anti-oxidation activity of NBIW. The mean (*SD*) ROS scavenging rate for hydroxy radicals generated by ultraviolet irradiation and aqueous hydrogen peroxide solution was 27.9 (0.21) in NBIW and 18.9 (2.81) in SBW, both of which were clearly higher than the 0.0 (4.85) in ultrapure water as a control. NBIW showed significantly higher hydroxy radical scavenging activity than SBW (*p* = 0.02^*^). The mean (*SD*) scavenging rate for superoxides was 20.0 (0.66) in NBIW and 19.7 (1.74) in SBW, both of which were also higher than in the control 0.0 (2.61).

### Clinical trial

To investigate the effect of NBIW in humans, we conducted a placebo-controlled, double-blind, randomized, parallel-group comparative trial with NBIW and control bath tablets. The ingredients of the NBIW and control bath tablets are shown in Table 1.

**Table 1:** Ingredients of the bath tablets.

Ingredients	NBIW	Control
Sodium bicarbonate	75%	0%
Sodium carbonate	5%	0%
Citric acid	15%	0%
Magnesium sulphate	0%	48%
Sodium sulphate	0%	48%
Other	5%	4%

*NBIW* neutral bicarbonate ionized water.

**Figure 3:**
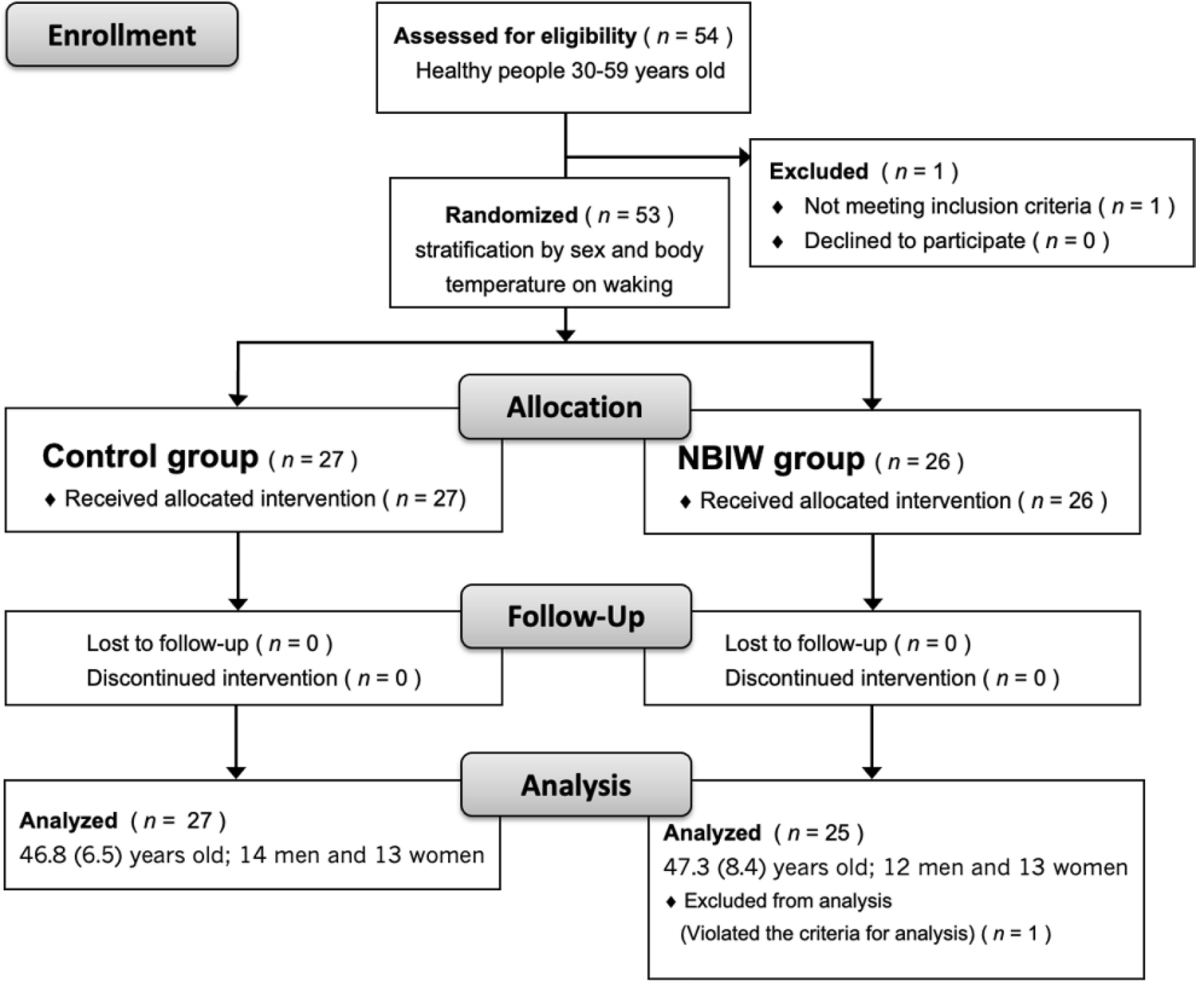
Flow diagram of randomized, double-blind, parallel-group study. *NBIW* neutral bicarbonate ion water.

Fifty-four participants (*n* = 27, 2 groups) were recruited; however, one person did not meet the selection criteria and was excluded, so the trial was performed in 53 participants. None of the participants discontinued or dropped out during the study, but one participant’s usage of the bath tablets met the exclusion criteria, so they were excluded from the analysis. Data from 25 participants in the NBIW group (mean [*SD*] age: 47.3 [8.4] years, 12 men and 13 women) and 27 participants in the control group (mean [*SD*] age: 46.8 [6.5] years, 14 men and 13 women) were available for analysis (Fig. 3). No intergroup differences were found in any demographic factors, including age, sex, alcohol intake, smoking, and exercise, or in the use rate of the study product or bathing time during the study period (Table 2).

**Table 2:** Background of the participants and use of bath tablets.

Items	Participants		
	NBIW	Control	*p* value
Number of participants	26	27	–
Male/female	13/13	14/13	0.893
Age, mean (*SD*), y	46.8 (8.5)	46.8 (6.5)	0.676
Drinks alcohol, yes/no	12/15	16/10	0.213
Current smoker, smoker/nonsmoker (of which smoked in the past)	1/24 (3)	0/27 (5)	0.304
Regular exercise, yes/no	9/18	13/13	0.218
Percentage use of bath tablets, mean (*SD*)	98.2 (4.2)	99.6 (1.5)	0.240
Bathing time, mean (*SD*), min	22.8 (7.4)	24.4 (6.6)	0.254

*NBIW* neutral bicarbonate ionized water.

No significant difference was found between control and NBIW groups in any item.

Age, use of test sample, bathing time: Wilcoxon rank sum test.

Other items: Chi-square test.

After 2 weeks, only the NBIW group showed a significant increase in body temperature on waking (*p* = 0.030*) and one hour after bathing (*p* = 0.002**) compared with before use of the test sample, but body temperature before bedtime was significantly increased in both groups compared with before use (NBIW: *p* = 0.005*, Control: *p* = 0.016*). After 4 weeks, body temperature was significantly higher than before the trial in both groups at all time points (Table 3).

**Table 3:** Body temperature.

Items	Sample	Before intervention	Intervention (week)			
			1	2	3	4
On waking	NBIW	36.12 (0.35)	36.22 (0.32)	36.23 (0.34)*	36.24 (0.34)*	36.29 (0.31)**
	Control	36.12 (0.3)	36.13 (0.28)	36.18 (0.29)	36.24 (0.29)**	36.22 (0.31)*
1 h after bathing	NBIW	36.42 (0.33)	36.47 (0.26)	36.55 (0.23)**	36.55 (0.23)*	36.57 (0.25)*
	Control	36.45 (0.3)	36.45 (0.44)	36.51 (0.25)	36.57 (0.28)*	36.57 (0.25)*
Before going to bed	NBIW	36.29 (0.25)	36.35 (0.28)	36.42 (0.21)*	36.39 (0.27)	36.44 (0.27)*
	Control	36.23 (0.25)	36.26 (0.29)	36.32 (0.24)*	36.38 (0.29)**	36.37 (0.29)**

*NBIW* neutral bicarbonate ionized water.

NBIW: *n* = 25, Control: *n* = 27.

Values are shown as mean (*SD*).

Within-group comparison: Significant differences were found compared with before the intervention (* *p* < 0.05, ** *p* < 0.01: Wilcoxon signed rank test).

Between-group comparison: No significant differences were found compared with the control group (Wilcoxon rank sum test).

A stratified analysis of the participants whose temperature on waking was below 36 °C showed a significant increase in temperature before going to bed in the NBIW group (*n* = 8) compared with the control group (*n* = 6) in both week 1 (*p* = 0.039^*^, Hedge’s *g* = 1.32 [95% CI: 0.19 to 2.59]) and week 2 (*p* = 0.002^**^, Hedge’s *g* = 1.55 [95% CI: 0.38 to 2.88]), but no intergroup differences were observed from week 3 onward (Table 4).

**Table 4:** Body temperature (stratified analysis by participants with body temperature below 36 °C on waking).

Items	Sample	Before intervention	Intervention (week)			
			1	2	3	4
On waking	NBIW	35.71 (0.26)	35.96 (0.41)*	35.95 (0.45)	35.97 (0.42)	36.08 (0.37)*
	Control	35.67 (0.14)	35.74 (0.09)	35.87 (0.27)	35.91 (0.26)*	35.81 (0.22)
1 h after bathing	NBIW	36.33 (0.42)	36.47 (0.23)	36.5 (0.24)	36.54 (0.21)	36.48 (0.18)
	Control	36.42 (0.30)	36.59 (0.31)	36.48 (0.27)	36.51 (0.29)	36.52 (0.26)
Before going to bed	NBIW	36.31 (0.31)	36.38 (0.25)^#^	36.43 (0.20)^##^	36.37 (0.28)	36.41 (0.23)
	Control	36.09 (0.22)	36.08 (0.14)	36.13 (0.15)	36.15 (0.26)	36.2 (0.17)*

*NBIW* neutral bicarbonate ionized water.

NBIW: *n* = 8, Control: *n* = 6.

Values are shown as mean (*SD*).

Within-group comparison: Significant differences were found compared with before the intervention (* *p* < 0.05: Wilcoxon signed rank test).

Between-group comparison: Significant differences between group (^#^ *p* < 0.05, ^##^ *p* < 0.01: Wilcoxon rank sum test).

Sample size was tested by noncentral t distribution before stratified analysis by using the week 2 temperatures before going to bed, and the power was found to be 0.739.

The score on the Japanese version of the Pittsburgh Sleep Quality Index (PSQI-J) was significantly lower in both the NBIW and control groups in Week 4 compared with baseline (NBIW:* p* = 0.003^**^, control:* p* = 0.002^**^), but no significant intergroup difference was observed (Table 5).

**Table 5:** Japanese version of Pittsburgh Sleep Quality Index (PSQI-J) scores.

Item	Sample	Before intervention	End point of intervention	*p* value	
				Within-group	Between-group
PSQI-J scores	NBIW	4.6 (2.0)	3.4 (1.2)**	0.003	0.249
	Control	3.9 (1.9)	3.0 (1.5)**	0.002	

*NBIW* neutral bicarbonate ionized water.

NBIW: *n* = 25, Control: *n* = 27.

Values are shown as mean (*SD*).

Within-group comparison: Significant difference compared with before intervention (**p < 0.01: Wilcoxon signed rank test).

Between-group comparison: No significant difference between-group (Wilcoxon rank sum test).

A psychological test with the simplified Profile of Mood States Second Edition-Adults Short (POMS2-A Short) showed a significant increase in vigor–activity (VA) in the NBIW group from before to after the intervention (Table 6), and the scores of all items related to the subjective symptom of cold intolerance were significantly lower in both groups after the intervention (Table 7). No serious adverse events or adverse reactions attributable to the use of the study product were observed.

**Table 6:** Profile of mood states 2nd edition-adult short (POMS2-A short) scores.

Items	Sample	Before intervention	End point of intervention	*p* value	
				Within-group	Between-group
AH (Anger-Hostility)	NBIW	41.4 (4.6)	42.8 (7.4)	0.336	0.246
	Control	42.7 (5.2)	43.0 (3.8)	0.624	
CB (Confusion-Bewilderment)	NBIW	42.4 (3.9)	43.2 (5.5)	0.292	0.659
	Control	44.6 (6.0)	43.3 (4.8)	0.189	
DD (Depression-Dejection)	NBIW	43.6 (3.3)	43.9 (3.4)	0.550	0.897
	Control	44.6 (4.3)	44.0 (3.8)	0.350	
FI (Fatigue-Inertia)	NBIW	41.1 (5.2)	41.7 (6.9)	0.886	0.692
	Control	42.4 (5.7)	40.3 (3.9)	0.057	
TA (Tension-Anxiety)	NBIW	42.2 (6.2)	42.3 (6.7)	0.983	0.883
	Control	44.9 (8.1)	42.9 (7.0)	0.054	
VA (Vigor-Activity)	NBIW	51.8 (8.7)	56.6 (10.0)	0.008**	0.706
	Control	55.9 (8.6)	56.3 (6.9)	0.667	
F (Friendliness)	NBIW	50.8 (11.5)#	52.1 (11.4)	0.296	0.790
	Control	56.0 (9.5)	53.2 (9.8)	0.185	
TMD (Total Mood Disturbance)	NBIW	41.2 (4.3)	40.9 (6.2)	0.732	0.854
	Control	42.1 (6.3)	40.7 (5.0)	0.095	

*NBIW* neutral bicarbonate ionized water.

Control: *n* = 27, NBIW: *n* = 25.

Values are shown as mean (*SD*).

Within-group comparison: A significant difference was found compared to before intervention (** *p* < 0.01: Wilcoxon signed rank test).

Between-group comparison: Before intervention, in “Friendliness” a significant difference was found compared with the control group (# *p* < 0.05); At the end point of intervention, no significant between group (Wilcoxon rank sum test).

**Table 7:** Subjective symptoms of cold intolerance.

Location of cold intolerance	Sample	Before intervention	End point of intervention	*p* value	
				Within-group	Between-group
Fingertips	NBIW	2.2 (1.1)	3.6 (1.1)**	0.00048	0.784
	Control	2.0 (1.1)	3.6 (1.4)**	0.00004	
Back of the hand	NBIW	3.0 (1.1)	4.5 (0.9)**	0.00036	0.902
	Control	2.7 (0.9)	4.4 (1.4)**	0.00006	
Whole hand	NBIW	2.5 (0.9)	4.1 (0.9)**	0.00013	0.490
	Control	2.4 (0.9)	3.9 (1.3)**	0.00009	
Abdomen	NBIW	3.0 (1.1)	4.6 (1.0)**	0.00009	0.165
	Control	2.9 (1.1)	4.2 (1.2)**	0.00025	
Lower back	NBIW	2.9 (1.2)	4.6 (1.1)**	0.00003	0.077
	Control	2.6 (1.0)	4.1 (1.2)**	0.00020	
Legs to toes	NBIW	1.4 (0.7)	3.3 (1.0)**	0.00003	0.421
	Control	1.4 (0.6)	3.1 (1.2)**	0.00004	
Whole body	NBIW	2.4 (0.9)	4.0 (0.9)**	0.00009	0.056
	Control	2.1 (0.9)	3.5 (1.2)**	0.00043	

*NBIW* neutral bicarbonate ionized water.

Control:* n* = 27, NBIW:* n* = 25.

Values are shown as mean (*SD*).

Within-group comparison: Significant differences were found compared with before use (**p < 0.01: Wilcoxon signed rank test).

Between-group comparison: No significant differences between group (Wilcoxon rank sum test).

## Discussion

In this study, we hypothesized that the mechanism of the blood flow enhancement effect of bathing in mineral-rich water is related to percutaneous absorption of CO_2_ and the subsequent formation of bicarbonate ions, i.e., we assumed that CO_2_ migrates to capillaries via the transappendageal route of the skin and exerts its blood flow-enhancing effect by directly acting on vascular endothelial cells as bicarbonate ions in the neutral pH environment in blood.

We reproduced the components of natural carbonated spring water rich in bicarbonate ions and referred to it as NBIW. We set the composition ratio of bicarbonate and citric acid in NBIW so that a solution with 2500 ppm of bicarbonate ions was obtained under a neutral pH environment when CO_2_ gas was generated by the neutralization reaction of sodium bicarbonate and citric acid. The results of our in vivo experiment in which mice were bathed in NBIW showed a significant increase in blood flow proportional to the increased blood level of bicarbonate ions resulting from NBIW bathing. The finding that the pH, PCO_2_, and bicarbonate volume of the NBIW used in the study showed no significant changes from before to during and after the study indicated that the concentration of bicarbonate ions in the solution remained stable.

**Figure 4:**
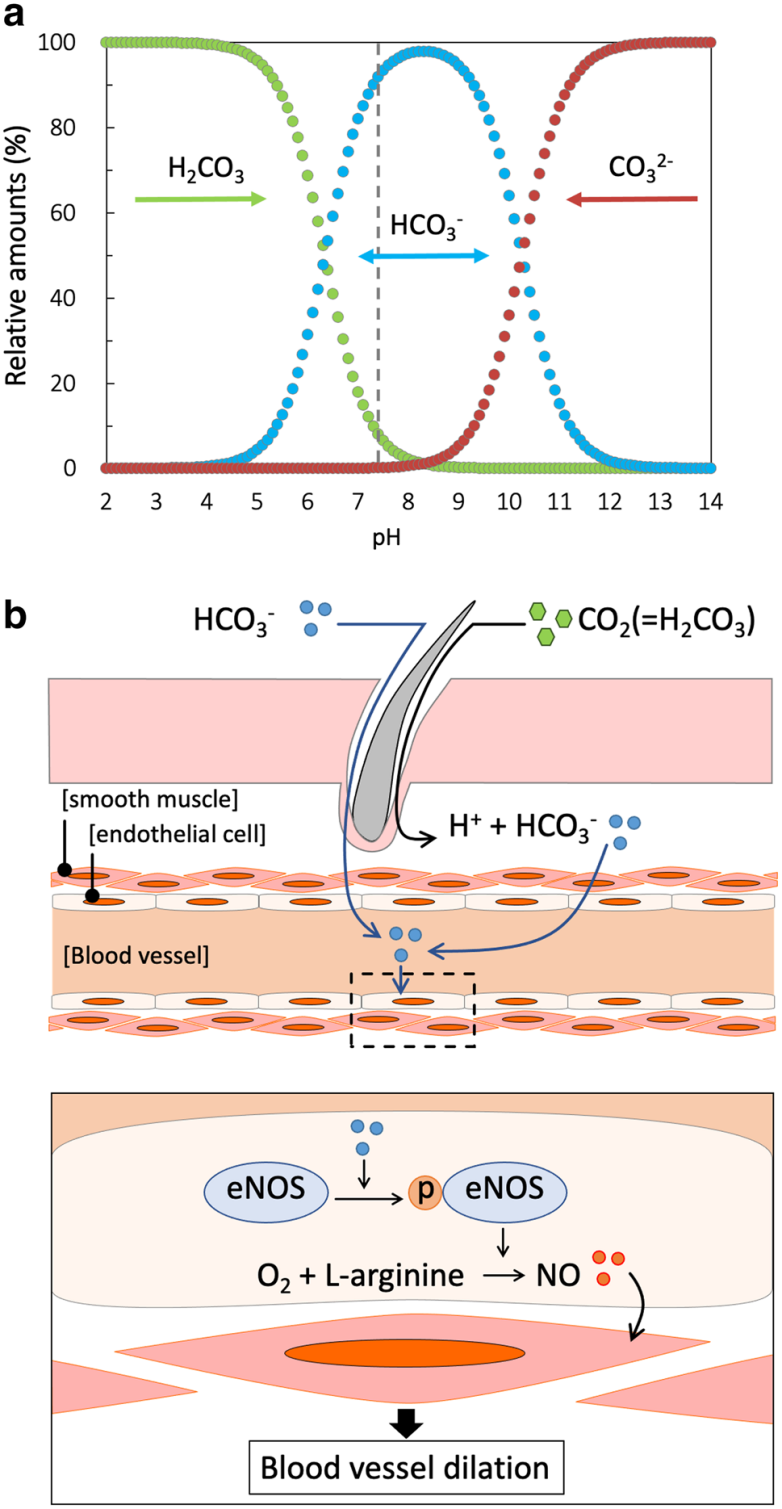
Schematic diagram of the underlying mechanism by which bicarbonate ions enhance blood flow. (**a**) Changes in the relative amounts of dissolved inorganic carbon (%) by pH. The pH of the neutral bicarbonate ion water (NBIW) was equivalent to that of blood, i.e., 7.4 (vertical dotted line). Among the dissolved inorganic carbon ions, bicarbonate ions (HCO_3_^−^) were most abundant. Modified from References 18 and 19. (**b**) Bicarbonate ions (HCO_3_^−^) and carbonic acid (H_2_CO_3_) are absorbed percutaneously, and carbonic acid is changed into bicarbonate ions in vivo. Bicarbonate ions that enter endothelial cells phosphorylate endothelial nitric oxide synthase (eNOS). Phosphorylated eNOS produces nitric oxide (NO), which relaxes the smooth muscle in the outside of endothelial cells, resulting in dilation of the vessel. *CO*_*3*_^*2−*^ carbonic ion, *eNOS* endothelial nitric oxide synthase, *H*^*+*^ hydrogen ion, *H*_*2*_*CO*_*3*_ carbonic acid, *HCO*_*3*_^*−*^ bicarbonate ion, *NO* nitric oxide, *p-eNOS* phosphorylated eNOS.

This study adjusted the ratio of sodium bicarbonate to citric acid so that the pH of NBIW was 7.4, i.e., equivalent to that of blood. At this pH level, dissolved inorganic carbonic acid is mostly present as bicarbonate ions (Fig. 4a). Moreover, when carbonic acid is percutaneously absorbed into blood, approximately 90% of the carbonic acid is known to be present as HCO_3_^–^^20^. Therefore, in our mice study we assume that bicarbonate ions were percutaneously absorbed during bathing with NBIW and that carbonic acid had less influence.

NO, a free radical with a short half-life that is synthesized in the human body, was discovered as a vascular endothelium-derived vasodilator^24^ that has a vasoprotective effect by causing vasodilation, retention of blood flow, suppression of neutrophil adhesion, suppression of platelet aggregation, active oxygen scavenging, and suppression of cell growth^25^. In vascula r endothelial cells, NO is synthesized by eNOS after it is activated via phosphorylation^26^. Given that the amount of eNOS, its phosphorylation level, and the amount of NO were increased in the mouse femoral vessels after NBIW bathing, our results show that NBIW bathing activates eNOS in vascular endothelial cells, increasing NO production and inducing vasodilation.

The results of our in vitro experiment in HUVEC also showed increased eNOS activity in the vascular endothelial cells cultured in a medium with NBIW. These results supported our hypothesis that bicarbonate ions percutaneously transferred into the capillaries during bathing act directly on the endothelial cells and induce NO production. The amount of bicarbonate ions in the NBIW used in this study was comparable to that in SBW. Although SBW increased the amount of blood circulation and NO production more than ultrapure water (control) during in vivo experiments, the response to SBW was weaker than that to NBIW. The blood flow-enhancing effect of SBW might have been weakened by oxidation of the produced NO. In the in vitro experiment, the hydroxy radical scavenging rate of NBIW determined by ESR was 1.5-fold higher than that of SBW. Therefore, prevention of oxidation of the produced NO by hydroxy radicals in NBIW might have contributed to an efficient increase in the amount of NO, which might explain the differences between NBIW and SBW in terms of NO production and the blood flow-enhancing effects.

To elucidate whether enhancing blood flow by regular bathing in bicarbonate ions is effective in increasing basal body temperature in humans and whether this elevated basal body temperature improves sleep and mental and physical conditions, the randomized, double-blind study in humans compared bath tablets containing sodium bicarbonate with control bath tablets containing sulfate salts; these salts do not generate bicarbonate ions. The results showed increased body temperature on waking, 1 h after bathing, and before going to bed in the NBIW group from week 2 onwards; although this effect was seen also in the control group, it occurred earlier in the NBIW group. Also, a stratified analysis of data from participants with a body temperature on waking below 36 °C showed a significant increase in body temperature in the NBIW group compared with the control group in weeks 1 and 2. In other words, NBIW bathing raised body temperature more quickly than bathing in a control solution containing magnesium sulfate and sodium sulfate, which are known spa ingredients.

The blood plays an important role as a heat exchanger in thermoregulation in humans, and decreased blood flow lowers body temperature^27,28^. The participants in our clinical trial were men and women aged 30 to 59 years with cold intolerance, and some of them presented symptoms of mild hypothermia. Thermoregulatory function declines with aging^29^, and one of the causes is an aging-associated decrease in production of NO, which has a vasodilator effect^30^. Some researchers reported that poorer thermoregulation with aging is related to suppression of mitochondria metabolism, increased ROS production, and accumulated oxidative damage to cells^31^. Thus, because aging is related to cold intolerance, the results of this study can be generalized only to middle-aged and older people. However, a limitation of this study is that it cannot clarify the effect of NBIW in people who do not have cold intolerance or in young people.

In vivo experiments showed that bathing in NBIW increased NO production and improved blood flow by enhancing endothelial function. Also, NBIW activated eNOS and ROS scavenging activity in vitro. Therefore, the finding in this study that bathing in NBIW improved hypothermia sooner than bathing in the control solution may be related to both direct endothelial activation and NO production, which protects cells from ROS. Recent reports showed that infective respiratory diseases were more likely to occur in persons with a lower body temperature because of the negative effects of cold on the immune system^32,33^. Additionally, 1 in 4 menopausal women in Japan have cold intolerance, which makes them susceptible to many diseases and conditions and may cause an imbalance of autonomic nervous system functions, such as sleep, and disturb their mental health^34^. However, cold intolerance is a problem that is often overlooked because it is difficult to define and a medical diagnosis and effective interventions are not available. Therefore, bathing in NBIW may have advantages for general health and subsequently may even lengthen life. This issue requires further study.

In conclusion, the results of our in vivo and in vitro experiments indicate that bicarbonate ions in NBIW are percutaneously absorbed during bathing and act directly on endothelial cells to increase NO production by phosphorylation of eNOS, leading to relaxation of vascular smooth muscle and enhanced blood flow. A schematic diagram of the underlying mechanism is shown in Fig. 4b.

Consequently, we suggest that the known effects of CO_2_-enriched spas on blood flow and vascular regeneration are also due to bicarbonate ions that form in the neutral pH environment of the blood after percutaneous absorption of CO_2_. In our clinical trial in middle-aged people, bathing in NBIW was confirmed to show a hyperthermal effect sooner than bathing in a control solution. The research presented here delivers new findings on the mechanism of enhanced blood flow after bathing in NBIW and clarifies its usefulness.

## Methods

### Mice

The experiments in mice were approved by the Tsurumi University Animal Experiment Committee (approval number: 20A020) and conducted according to the related guidelines, laws, and regulations. The study is reported in accordance with ARRIVE guidelines (https://arriveguidelines.org).

Male BALB/c mice aged 11 to 14 weeks were reared in a husbandry facility with controlled temperature and humidity under a 12-h light cycle. The mice had free access to feed and water. Randomization was not used to allocate animals to groups. No data were obtained from animals that died during the experiment. Confounders were not controlled, and all study investigators were aware of group allocation during the experiments, assessment of outcome, and data analysis.

### NBIW bathing 

Sodium bicarbonate was diluted in ultrapure water at 3.8 mg/mL, and citric acid was added to the solution at 0.5 mg/mL to obtain a solution that had a neutral pH and 2500 mg/L of bicarbonate ions. Ultrapure water and SBW at 3.8 mg/mL were used as controls. The bicarbonate ion concentration was determined by titration with sulfuric acid. The BALB/c mice were anesthetized with 3 types of mixed anesthetic agents (0.3 mg/kg of medetomidine, 4.0 mg/kg of midazolam, and 5.0 mg/kg of butorphanol)^35^ and bathed in NBIW (n = 6) or control solution at 37 °C for 20 min. For the NOS inhibitory experiment, L-NAME 100 mg/kg was injected intraperitoneally into 4 mice 1 h before bathing. After bathing, blood flow in the mice was determined by the laser doppler blood flowmeter RBF-101 (Pioneer Corporation).

The other 2 mice were bathed under anesthesia and euthanized by cutting the carotid vein with a 5-mm Glodenrod Animal Lancet (MEDIpoint, Inc.), and the released blood was collected in a 1.5-mL centrifuge tube. The blood gas analyzer GASTAT-navi (Techno Medica Co., Ltd.) was used to measure the partial pressure of oxygen, partial pressure of carbon dioxide, pH, and bicarbonate levels of the collected blood. The femoral arteries and veins of the mice were also isolated and stored at − 80 °C. The isolated vessels were homogenized in purified water, and the amount of NO was measured by the NO_2_/NO_3_ Assay kit-FX (Fluorometric) 2,3-Diaminonaphthalene Kit (Dojindo Laboratories).

The pH, PCO_2_, and bicarbonate in the NBIW used for the study were measured before, during, and after the experiment.

### Western blot analysis

The vessels isolated from the mice were homogenized in radioimmunoprecipitation assay (RIPA) buffer containing protease inhibitor and phosphatase inhibitor, and the tissue lysate was used for Western blot analysis with anti-eNOS antibody (M221; ab76198, 1:1000, Abcam plc), anti-eNOS (phospho S1177) antibody (EPR20991; ab230158, 1:1000, Abcam plc), and anti-β-actin antibody (HRP-60008, 1:5000, Proteintech). eNOS, p-eNOS and β-actin in the samples were detected by C-DiGit^®^ Blot Scanner (LI-COR Inc.) on the basis of the chemiluminescence intensity generated by the ECL Prime Western Blotting Detection Reagent (Cytiva) and were quantitatively assessed by Image Studio™ (LI-COR Inc). After SDS-PAGE, gels were separated at each target molecule range to normalize eNOS and p-eNOS expression level by β-actin, a housekeeping protein. Each gel was then transferred to a polyvinylidene difluoride membrane and hybridized with primary antibody for eNOS and p-eNOS or β-actin.

### In vitro experiments in HUVEC

HUVEC were starved for 6 h in a medium not containing serum or VEGF supplement (KBM VEC-1; Kohjin Bio Co., Ltd). NBIW was diluted 1,000,000-fold in the culture medium so that the bicarbonate ion content became 2.5 ppb. After 5 min, cell lysate was obtained in ultrapure water or RIPA buffer, and the amount of NO and eNOS phosphorylation activity was evaluated as described above.

### ESR analysis

The ROS scavenging activity of NBIW was determined by ESR. Superoxide generated by xanthine/xanthine oxidase, a major ROS, and a hydroxy radical generated by UV-irradiated hydrogen peroxide solution were trapped by 5-(2,2-dimethyl-1,3-propoxy cyclophosphoryl)-5-methyl-1-pyrroline N-oxide (CYPMPO) and determined as a CYPMPO adduct^36^. NBIW at a concentration 100-fold higher than that used for the mouse bathing experiment was prepared, and its ROS scavenging activity was compared with that of SBW at a concentration 100-fold higher than that used in the mouse bathing experiment and that of ultrapure water. Each condition was repeated 3 times, and the data were analyzed.

### Randomized, double-blind, parallel-group comparison study

The clinical study was performed in compliance with the Declaration of Helsinki and CONSORT statement and was approved by the Chiyoda Paramedical Care Clinic Ethics Review Committee (IRB number: KRK171C1; approval number: HTT18C1) and registered with the UMIN Clinical Trials Registry (UMIN000031026) on 29/06/2018. All participants provided written informed consent to participate in the study. The study was conducted between December 2016 and April 2017, and no major changes were made to the protocol after the start of the study. Data were collected at the Chiyoda Oral Healthcare Clinic and Chiyoda Paramedic Care Clinic, and statistical analysis of the data was performed by CPCC Company Limited, a contract research organization.

Participants consisted of male and female volunteers between 30 and 59 years of age with a subjective symptom of cold intolerance who fulfilled the inclusion criteria and did not meet the exclusion criteria. Inclusion criteria were subjective symptoms of cold intolerance or consistently low body temperature, menopause or a stable menstrual cycle in women, and ability to bathe daily during the study period and to provide written informed consent. Exclusion criteria included regular intake of medicine, regular consumption of foods that may improve blood flow and body temperature, history of serious illness or skin conditions, and pregnancy or breastfeeding. The participants were divided into 2 groups by block randomization on the basis of sex and body temperature on waking. The person responsible for allocating assigned participants to be given either NBIW tablets containing bicarbonate ions as the main ingredient or control tablets containing magnesium sulphate and sodium sulphate. Table 1 shows the composition of NBIW and control tablets. Both tablets were designed as white tablets of 15 g each that were indistinguishable. The randomization code was not disclosed to participants or the people responsible for collecting and analyzing trial data until the end of analysis. Participants were tested during a 1-week pre-observation period and a 4-week intervention trial. They were instructed to dissolve 4 tablets in a bath with hot water at a temperature below 41 °C once daily for 4 weeks and to soak in the water for at least for 15 min. They were also told to not make significant changes to their lifestyle during the study period. Primary outcome endpoints were body temperature on waking, sleep quality assessed by the Japanese version of Pittsburgh Sleep Quality Index (PSQI-J)^37,38^, and score on the simplified POMS-2 for adults^39,40^. Secondary outcomes were body temperature before lunch, before supper, before bathing, 1 h after bathing, and before going to bed and the presence or absence of a subjective symptom of cold intolerance. Participants were given a logbook and instructed to use it daily to record the items specified in the protocol, such as use of bath tablets, time of bathing, and body temperature upon waking and at bedtime, from one week before to the end of the intervention.

### Statistical analysis

Statistical analyses were performed with Mac statistical analysis (ESUMI Co., Ltd.) and StatPlus:mac (AnalystSoft Inc.). For the animal experiments, comparisons of 2 independent groups were performed by Student’s t test for parametric data and the Mann–Whitney *U* test for non-parametric data. For the clinical study, Wilcoxon rank sum test or Chi-square test was used for comparisons of 2 independent groups and Wilcoxon signed rank test, for intragroup comparisons before and after the study. Hedge’s *g* with the 95% CI was calculated as an index of impact of the effect^41^.

## Supplementary Information

Supplementary Information.

## Data Availability

All study data are available from the corresponding author.
