# Supplementary material for: The effects of bathing in neutral bicarbonate ion water
Source: Sci Rep. 2021 Nov 8;11:21789. doi: 10.1038/s41598-021-01285-4 (PMC8576025; doi:10.1038/s41598-021-01285-4)
Supplement: Supplementary file 1 — Supplementary Information. [file 41598_2021_1285_MOESM1_ESM.pdf]

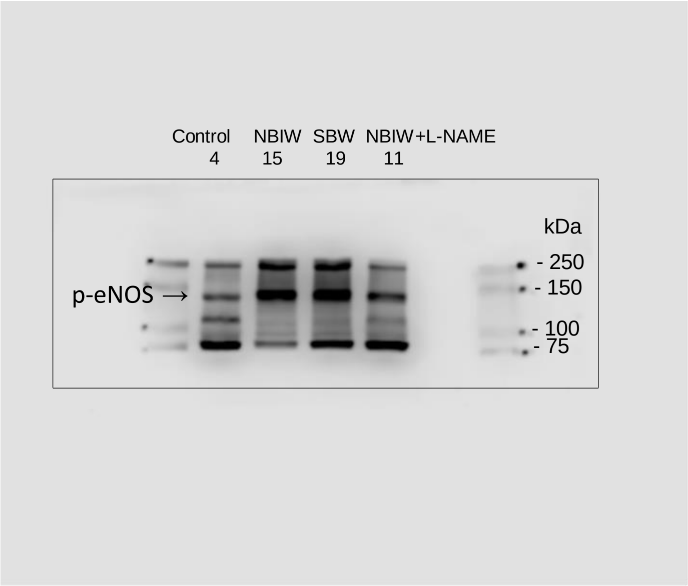

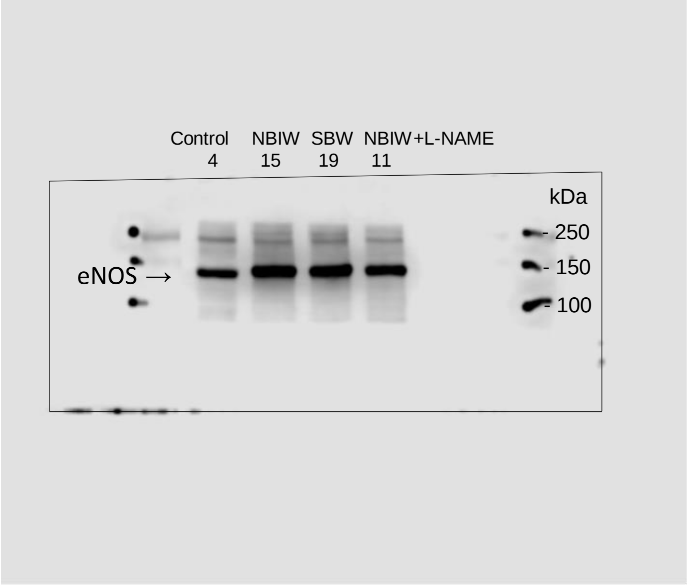

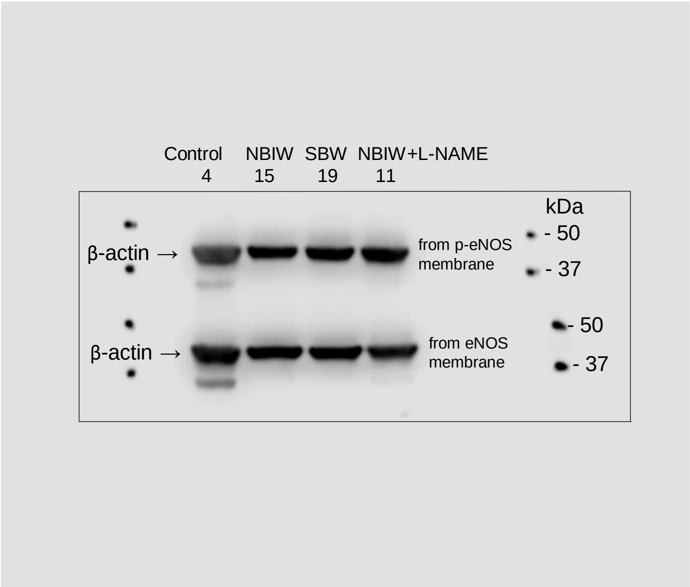

Full-length blots for Figure 1d.

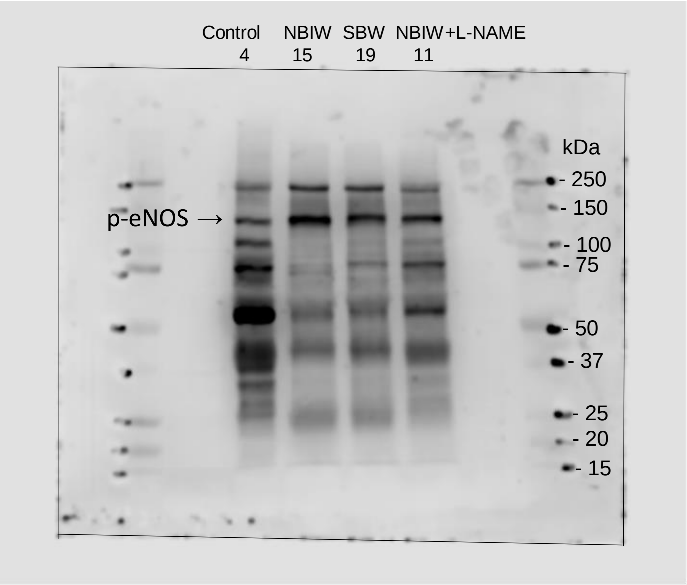

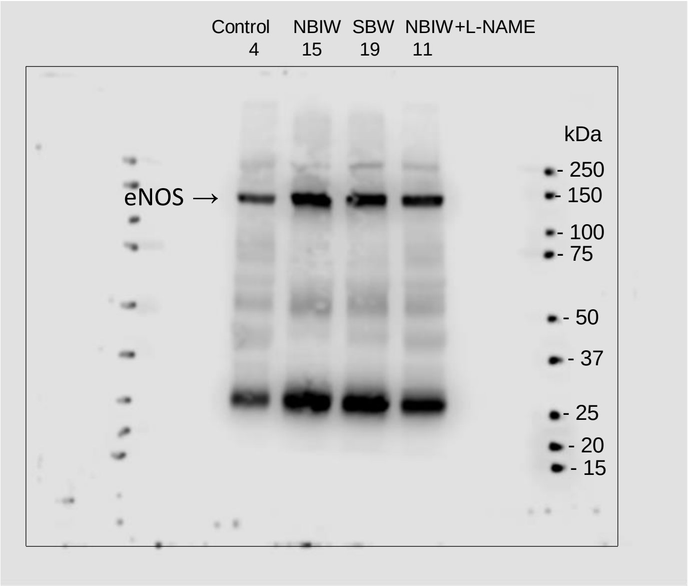

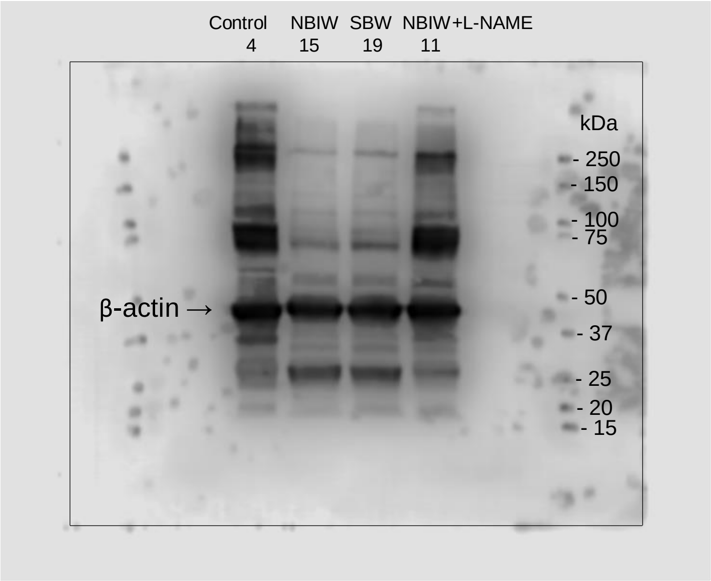

Unprocessed images of full-length blots for Figure 1d.

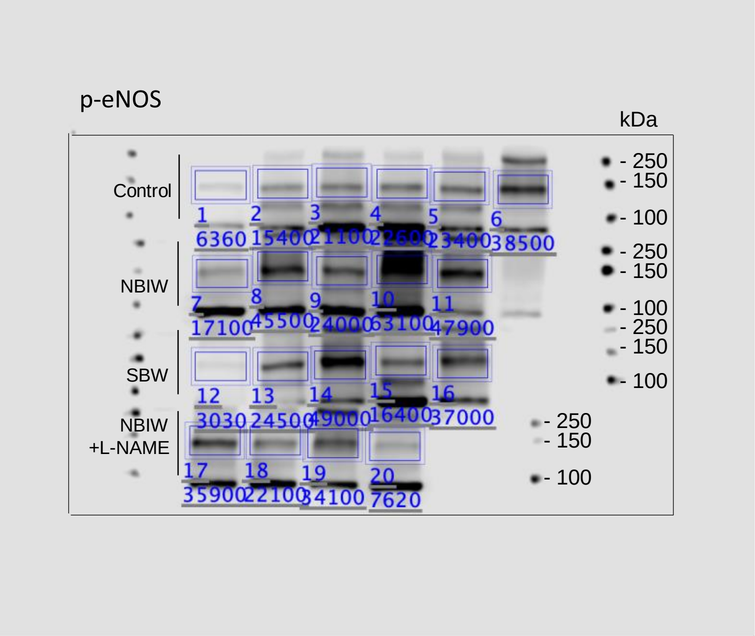

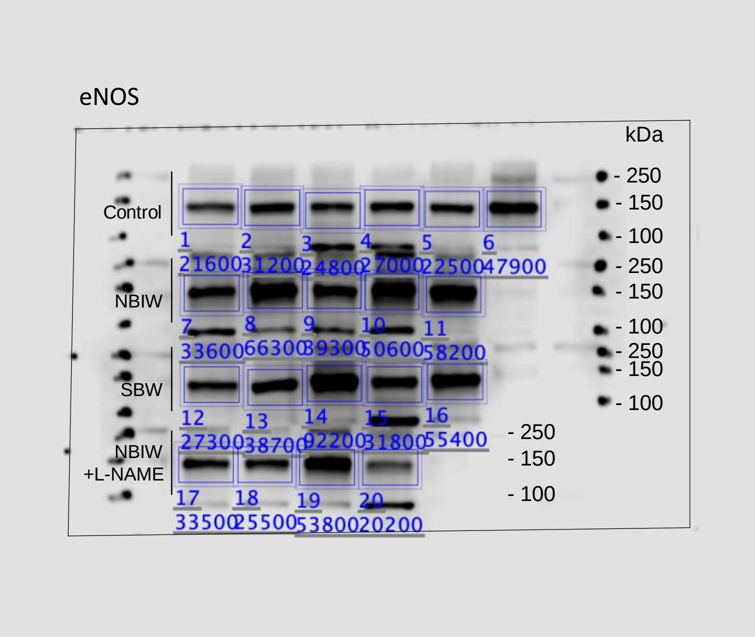

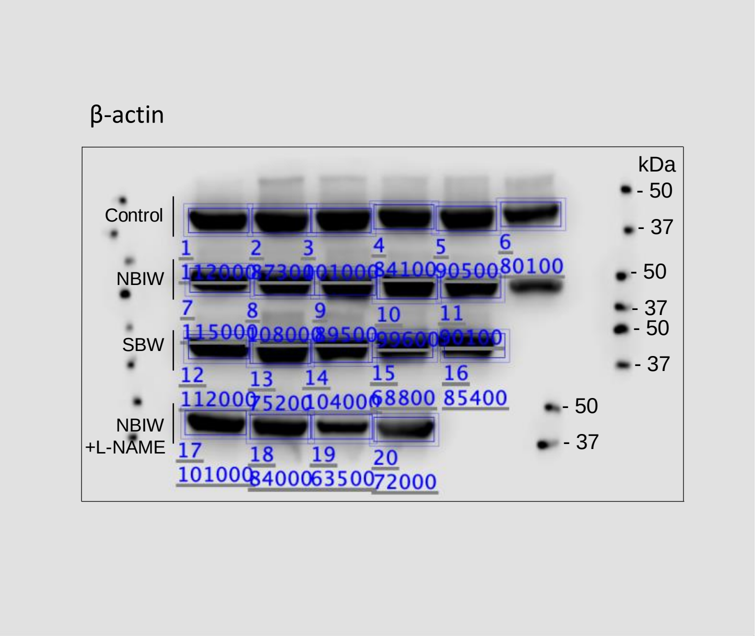

Full-length blots for Figures 1e, f, and g.

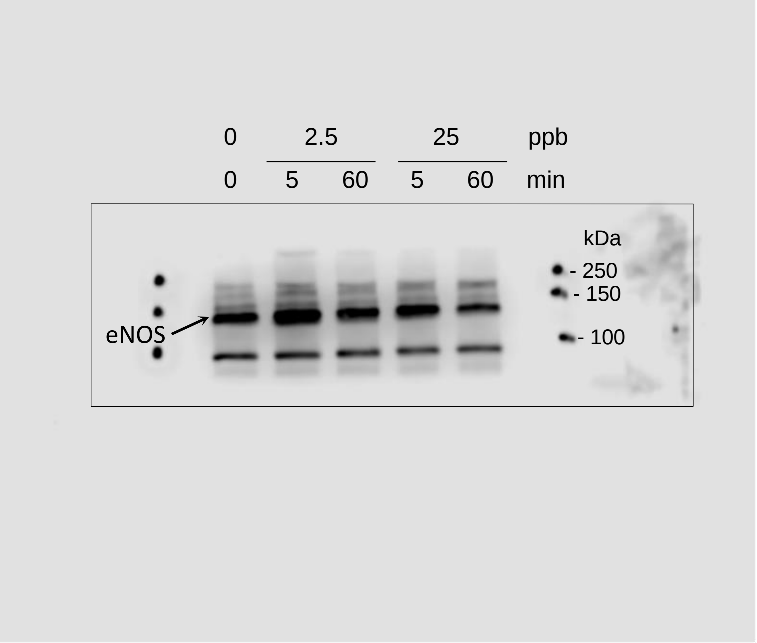

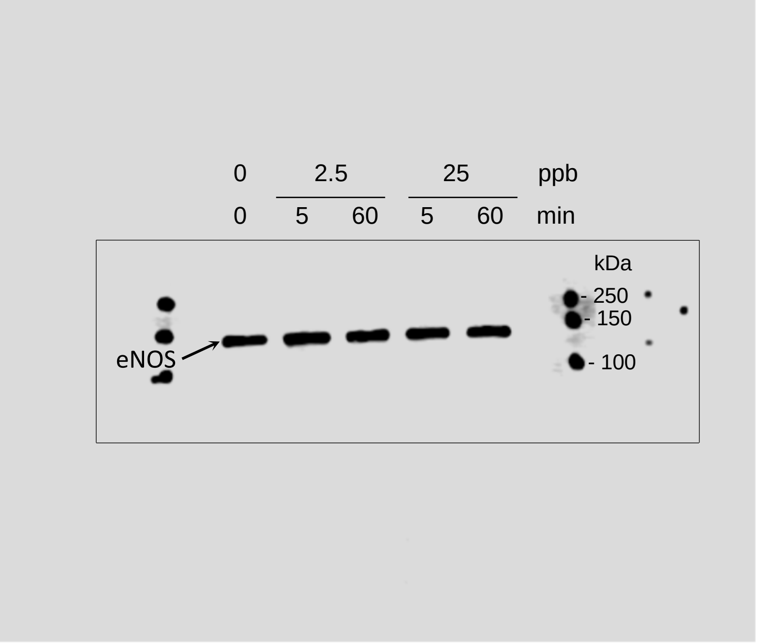

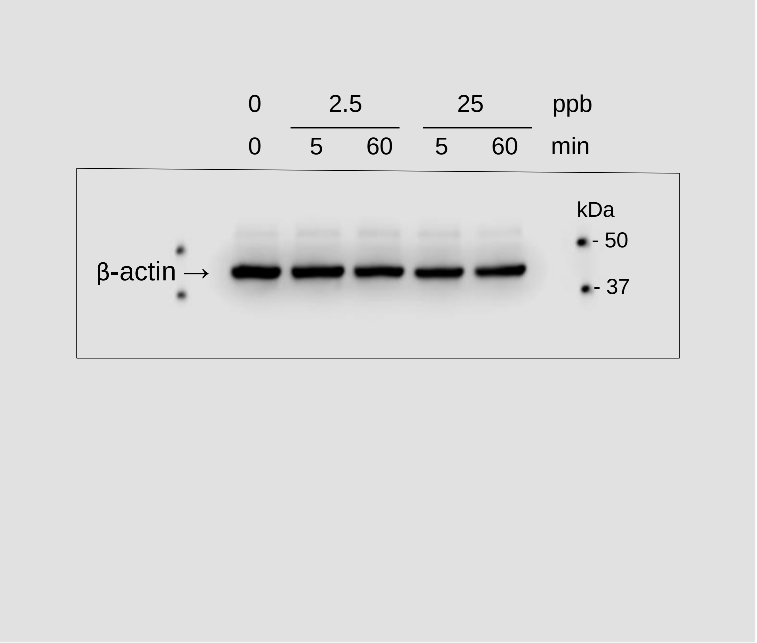

Full-length blots for Figure 2a.

Changes in PCO_2, pH, and HCO_3^- over time (average of triplicate measurements)

	PCO_2（Torr)			pH			HCO_3^- (mg/L)		
	Initial	30min.	1H	Initial	1H	3H	Initial	1H	3H
NBIW	57.3	32.4	58.4	7.33	7.37	7.40	2456.27	2427.80	2442.03
SBW	6.2	7.0	6.0	8.36	8.40	8.40	2677.90	2702.30	2639.27
Control	＜5.0	＜5.0	＜5.0	6.67	6.43	7.07	0.19	0.18	0.18

NBIW: sodium bicarbonate 3.8 g + citric acid 0.5g /MilliQ 1L

SBW: sodium bicarbonate 3.8g /MilliQ 1L

Control: MilliQ
